# Sleep and cognition in Hispanic/Latin American adults: A systematic review

**DOI:** 10.1002/alz.71512

**Published:** 2026-05-22

**Authors:** Kasey J. Escamilla, Alexa S. Gonzalez, Michelle N. Martinez, Candice A. Alfano, Luis D. Medina

**Affiliations:** ^1^ Department of Psychology University of Houston Houston Texas USA; ^2^ Department of Psychology Stony Brook University Stony Brook New York USA; ^3^ Department of Psychiatry and Behavioral Sciences Baylor College of Medicine Houston Texas USA

**Keywords:** adult, aging, cognitive dysfunction, Hispanic, sleep

## Abstract

Sleep disturbance is associated with increased risk for cognitive impairment. However, previous research is primarily based on non‐Hispanic White individuals, despite higher rates of sleep problems and disorders among Hispanic/Latin American individuals (H/Ls). The present study reviews the current literature on associations between sleep and cognitive function in H/L adults. Following Preferred Reporting Items for Systematic Reviews and Meta‐Analyses (PRISMA) guidelines, we examined literature that (1) examined the association between sleep and cognitive decline in H/Ls, and (2) had a sample with at least 5% H/Ls. We found equivocal evidence of associations between sleep parameters and cognitive performance in H/Ls, with the most compelling evidence supporting a relationship between long sleep and lower cognitive function. Heterogeneity in operationalization and measurement likely contributes to these equivocal findings. Future cross‐sectional, experimental, and longitudinal research in H/Ls is needed to address major knowledge gaps.

## Highlights

- Our review has identified equivocal evidence of associations between various sleep parameters (e.g., sleep duration, insomnia, sleep‐disordered breathing, and sleep onset latency) in Hispanic/Latin American individuals (H/Ls). There was especially compelling evidence supporting a relationship between long sleep and lower cognitive function.
- However, there was great heterogeneity in the operationalization and measurement of the sleep parameters included in the review.
- Such heterogeneity likely contributed to the equivocal findings reported in this review and overall low quality evidence linking sleep and cognitive aging in this population.

## 1 BACKGROUND

Due to advancements in healthcare and nutrition, the aging population is expanding at a rapid pace. By the year 2050, the population of older adults is expected to double in size.^1^ Aging is associated with various physiological changes, including alterations in sleep patterns and quality. For instance, normative sleep‐related changes observed in older adults include decreased sleep duration (total time spent asleep), less time in deep sleep (i.e., slow wave sleep), increased sleep disruptions throughout the night, and reduced overall sleep quality compared to younger adults.^2^, ^3^ Because poor quality sleep is closely associated with deficits in neuropsychological functioning,^4^ these normative changes in sleep may, in part, contribute to changes in cognition in older adults.

Age‐related alterations in sleep patterns and quality are a function of other co‐occurring changes affecting neuroendocrine, homeostatic, and circadian systems, among others.^5^ For example, decreased homeostatic sleep pressure in older adults is thought to contribute to reduced total sleep and increased sleep fragmentation, advances in circadian phase alter the timing of sleep periods, and levels of various neurotransmitters involved in arousal (e.g., dopamine, serotonin, and norepinephrine) diminish with age. Accordingly, certain sleep disorders are more common in older adults compared to younger adults, including insomnia and obstructive sleep apnea (OSA).^2^, ^6^ Insomnia is characterized by chronic problems with falling or staying asleep, or non‐restorative sleep,^7^ while OSA, a form of sleep‐disordered breathing (SDB), is a serious condition characterized by partial or complete cessation of breathing that disrupts sleep continuity and quality.^2^

Poor sleep health, even in the absence of a clinical sleep disorder, is well known to impact multiple aspects of human performance, including physiological, hormonal, behavioral, and cognitive health. The most acute and profound effects of inadequate or poor‐quality sleep are routinely observed on cognitive performance.^8^, ^9^ Previous research suggests that, when healthy adults achieve less than 7 hours of sleep, they perform more poorly on cognitive tests for alertness, reaction time, memory, and decision‐making.^9^ Additionally, healthy adults experiencing chronic sleep restriction (i.e., four or more days with 7 hours or less of sleep) experience cumulative adverse effects on neurobehavioral functions.^10^ The recovery of such neurobehavioral functions has a linear relationship with increasing sleep duration in healthy adults experiencing chronic sleep restriction.^11^ Evidence also suggests that sleep disturbances may be closely associated with increased risk for cognitive disorders, like Alzheimer's disease and related dementias.^3^, ^12^

However, much of the research examining the relationship between sleep and cognition has been conducted on majority non‐Hispanic White populations; as such, research in minoritized populations, such as Hispanic/Latin American individuals (H/Ls), is limited. The limited cultural and linguistic diversity of research samples limit the generalizability of study findings as we cannot assume trends observed in largely non‐Hispanic White individuals would be the same for other racial and ethnic populations. This is especially the case when considering potential cultural factors that influence sleep, such as acculturation, psychosocial stressors, attitudes, beliefs, and specific cultural practices.^13^, ^14^ For instance, H/Ls are more likely to take naps during the day in accordance with the *siesta* custom from Latin American countries, where individuals take a nap following lunch to rest during the hottest part of the day. A study by Taub^15^ surveyed a sample of community‐dwelling adults in Hermosillo, Mexico, and reported that the average length of daytime sleep in this sample was over an hour. Previous literature has shown benefits to short naps (10–20 min), including improvements in mood and cognitive performance.^16^ However, longer naps that exceed 30 min are associated with greater sleep inertia, which is associated with grogginess, confusion, and poorer cognitive performance.^16^ Furthermore, differences in knowledge, attitudes, and beliefs about sleep may influence sleep hygiene practices.^17^

H/Ls, individuals with origins from Spanish‐speaking countries and/or Latin America, are the largest and one of the fastest‐growing demographic groups in the United States. Compared to non‐Hispanic White individuals, H/Ls are at increased risk for cognitive decline, psychological stressors, obesity, diabetes, hypertension, and cardiovascular disease.^13^, ^18^, ^19^ Additionally, the positive relationship between serious psychological distress and sleep disturbances (e.g., trouble falling asleep, trouble staying asleep, feeling rested upon awakening, and use of sleep medication) tends to be stronger in H/L adults than in non‐Hispanic White adults.^18^ H/Ls also experience a high incidence of short sleep, poor quality sleep, and several sleep disorders.^20^ Given these findings, it is critically important to elucidate the impact of modifiable risk factors, like sleep disturbances, on cognitive functioning in H/Ls. Along these lines, this systematic review aimed to examine available literature on the associations between various sleep parameters (e.g., sleep duration, SDB, and insomnia) and cognitive function in H/L adults.

## 2 METHODS

The present systematic review was conducted in two stages in accordance with the Preferred Reporting Items for Systematic Reviews and Meta‐Analyses (PRISMA) guidelines.^21^ Stage 1 consisted of a review across five databases. This search sought to compile a list of peer‐reviewed journal articles that examined sleep disturbances and cognition. Stage 2 consisted of a full‐text screening of the remaining articles and was conducted by two investigators. During this process, we identified articles that (1) examined the association between at least one sleep variable and cognitive performance and (2) had a sample consisting of at least 5% H/L adults. Given the anticipated dearth of literature including H/L adults, we used a 5% cutoff to help gather relevant articles without being too restrictive. Conference abstracts, theses, dissertations, and book chapters were excluded from the review.

### 2.1 Inclusion and exclusion criteria

We included records that (1) discussed or investigated at least one aspect of sleep and (2) included samples composed of H/L adults (ages 18 years and older). With consideration of the limited number of publications on this topic, we did not incorporate any exclusionary criteria regarding publication date or study type to ensure we maximized our search. Records were reviewed independently by two reviewers (K.E. and A.G.); discrepancies were resolved with the help of a third reviewer (M.M.).

### 2.2 Information sources

We used five academic databases for our search: EMBASE, PsycInfo, PubMed, Scopus, and Web of Science. These databases were chosen given their inclusion of peer‐reviewed journals with relevant topics in the psychological sciences focusing on sleep and cognitive functioning.

### 2.3 Search strategy

The following key terms were used to conduct our search:

**Table:** 

Sleep* or somnolence or jetlag or “shift work” or jetlag or “jet lag”	AND	Cognit* or memory or “brain health” or “Alzheimer's disease” or dementia	AND	Hispanic or Latin* or “Hispanic American” or Chican*	AND	“Older adults” or “aging adults”

Various efforts were made to maximize the number of search results given the limited research with H/L communities. Along these lines, we incorporated terms such as “jet lag” and “shift work” since articles covering these topics may address sleep disturbances. We utilized asterisks as a wildcard symbol for words such as “Latin” and “sleep” to efficiently broaden our search to include words that began with the same letters, such as “Latino/a/e,” “Latinx,” “sleepy,” “sleepiness,” and to capture the various sleep factors that begin with the word “sleep” (e.g., sleep onset latency [SOL], sleep apnea, sleep efficiency, and sleep stages). Although our review primarily focused on cognitive function, we included “Alzheimer's disease” and “dementia” in our key terms to broaden the search results. Given that cognitive aging spans decades and has multiple contributing factors, we used the age of 18 as a cutoff to capture a wider net of adult ages.

### 2.4 Data collection process

**FIGURE 1:**
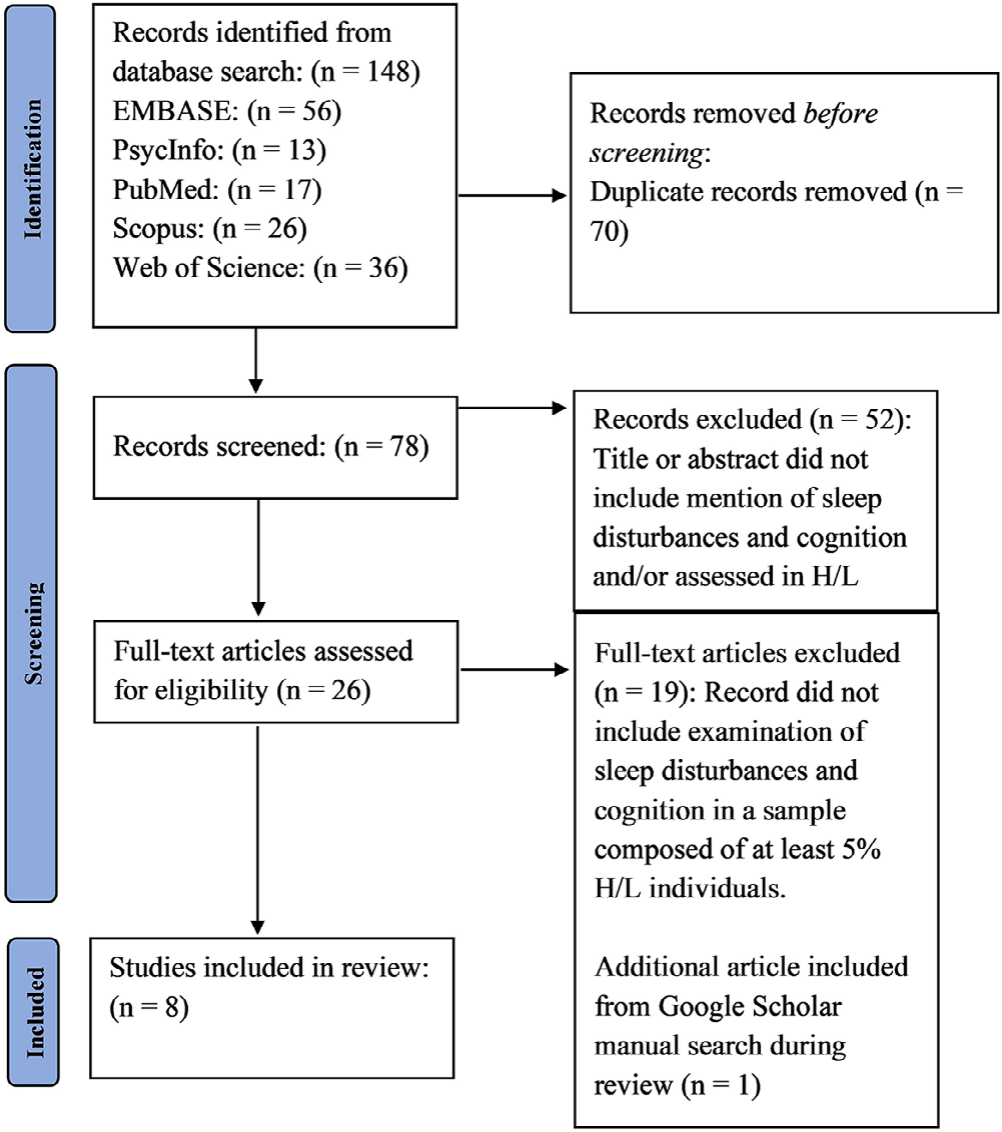
PRISMA diagram for database search. PRISMA, Preferred Reporting Items for Systematic Reviews and Meta‐Analyses

The screening process was conducted in two phases: (1) screening titles and abstracts to assess relevance to the inclusion and exclusion criteria and (2) screening full‐text articles. Specifically, a preliminary read of the full‐text articles was performed to compare the content with the inclusion and exclusion criteria. Reasons for exclusion included: (1) no examination of the association between sleep and cognition in H/Ls, and (2) the sample did not consist of at least 5% H/Ls. Details regarding the number of articles excluded at each phase can be found in Figure 1, which provides a summary of the screening process.

### 2.5 Data items

From the studies selected for the review, the following information was extracted: the types of sleep problem or parameter examined (e.g., sleep duration, insomnia symptoms, SOL), the measures used to assess sleep parameters and cognition, sample size, source of recruitment, average age and range, and language of administration. We also collected information about the key findings from each study pertaining to H/L adults. We primarily noted results discussing the relationship between sleep parameters and cognitive function.

### 2.6 Quality assessment across studies

The National Heart, Lung, and Blood Institute's Quality Assessment Tool for Observational Cohort and Cross‐Sectional Studies was used^22^ to examine the quality of the studies reviewed. Two individuals (K.E. and A.G.) separately evaluated the studies using the quality assessment tool and convened to discuss discrepancies with the help of a third reviewer (M.M.). Information regarding the assessment for risk of bias is available in Table 1.

**TABLE 1:** Risk of bias assessment

Criteria	Agudelo et al. 2021	Gallagher et al. 2022	Kaur et al. 2021	Ramos et al. 2013	Ramos et al. 2015	Ramos et al. 2016	Ramos et al. 2020	Ramos et al. 2021
1. Was the research question or objective in this paper clearly stated?	Yes	Yes	Yes	Yes	Yes	Yes	Yes	Yes
2. Was the study population clearly specified and defined?	Yes	Yes	Yes	Yes	Yes	Yes	Yes	Yes
3. Was the participatio*n* rate of eligible persons at least 50%?	Yes	Yes	Yes	Yes	Yes	Yes	Yes	Cannot determine
4. Were all the subjects selected or recruited from the same or similar populations (including the same time period)? Were inclusion and exclusion criteria for being in the study prespecified and applied uniformly to all participants?	Yes	Yes	Yes	Yes	Yes	Yes	Yes	Yes
5. Was a sample size justification, power description, or variance and effect estimates provided?	No	No	No	No	No	No	No	No
6. For the analyses in this paper, were the exposure(s) of interest measured prior to the outcome(s) being measured?	Not reported	Yes	Yes	No	Not reported	Yes	Yes	Yes
7. Was the timeframe sufficient so that one could reasonably expect to see an association between exposure and outcome if it existed?	Cannot determine	Yes	Yes	No	Cannot determine	Yes	Yes	Yes
8. For exposures that can vary in amount or level, did the study examine different levels of exposure as related to the outcome (e.g., categories of exposure, or exposure measured as continuous variable)?	Yes	No	Yes	Yes	Yes	Yes	Yes	Yes
9. Were the exposure measures (independent variables) clearly defined, valid, reliable, and implemented consistently across all study participants?	Yes	Yes	Yes	Yes	Yes	Yes	Yes	Yes
10. Was the exposure(s) assessed more than once over time?	Yes	Yes	No	No	Not reported	Not applicable	No	No
11. Were the outcome measures (dependent variables) clearly defined, valid, reliable, and implemented consistently across all study participants?	Yes	Yes	Yes	Yes	Yes	Yes	Yes	Yes
12. Were the outcome assessors blinded to the exposure status of participants?	Not applicable	Not applicable	Not applicable	Not applicable	Not applicable	Not applicable	Not applicable	No
13. Was loss to follow‐up after baseline 20% or less?	No	Yes	Cannot determine	No	Not applicable	Not applicable	Cannot determine	Not applicable
14. Were key potential confounding variables measured and adjusted statistically for their impact on the relationship between exposure(s) and outcome(s)?	Yes	Yes	Yes	Yes	Yes	Yes	Yes	Yes
Quality rating	Good	Good	Fair	Fair	Fair	Good	Fair	Fair

## 3 RESULTS

### 3.1 Study selection

As shown in Figure 1, after conducting the initial search, we obtained a total of 148 records. After removing duplicates, 78 unique records were available for subsequent screening of titles and abstracts. Fifty‐two of these records were excluded as the studies either lacked any mention of sleep disturbances in the title or abstract, or they did not assess sleep disturbances in H/Ls. We conducted a preliminary review of the full text of the remaining 26 articles. During this process, 17 articles were removed that did not meet the inclusion/exclusion criteria. During review of the selected articles, one additional article was identified that met the inclusion/exclusion criteria and was subsequently included in the final review. Additionally, after a full‐text review of 10 studies that met inclusion criteria, it was revealed that the majority of studies had a high proportion of H/Ls, where the average percentage across the 10 studies was 83% (9.81% –100%). One study^23^ reported having a sample of 5% H/Ls and was initially included in this review. However, upon further evaluation of the analytic sample, the study was ultimately excluded due to its analytic sample being comprised of only 4.3% H/Ls. Another study was conducted in Sao Paulo Brazil.^24^ Given that the remainder of the studies were conducted in North America, this study was removed from the final list out of consideration for differences in environmental factors that may influence differences in sleep trends across the different populations. Thus, the final list for the synthesis comprised of eight articles.

#### 3.1.1 Study characteristics

The characteristics for each of the eight articles reviewed are outlined in Table 1, categorized according to the primary sleep problem or parameter examined in each of the respective studies. Despite using an age cutoff of 18, the samples of the final list of articles in this review was ultimately 40 years and older, with a total of 30,614 participants. Seven out of eight studies had a largely H/L sample, with most samples being at least 68% H/L. Five of these studies had a fully H/L sample. However, the samples of these studies all originated from the same parent cohort, Hispanic Community Health Study/Study of Latinos (HCHS/SOL). Thus, there is potential overlap between these samples.

Each of the studies is discussed within the subheadings of sleep parameters, and a summary of the sample demographics, sleep parameters examined, cognitive measures used, language of administration, and key findings is reported.

### 3.2 Risk of bias

A complete description of quality ratings for all studies can be found in Table 2. No significant biases were identified during quality assessment across studies using the National Heart, Lung, and Blood Institute's Quality Assessment Tool for Observational Cohort and Cross‐Sectional Studies.^22^ Five studies were given a “fair” rating, and the remaining three studies were given a rating of “good.”

**TABLE 2:** Study characteristics

Journal article	Sample demographics	Sleep parameters	Cognitive measures	Language of administration	Key findings
Sleep duration					
Ramos et al., 2013	927 participants (ages 40 or over, mean age of 75 years ± 9 years) from the Northern Manhattan Study cohort, 68% H/L	Sleep duration (Epworth Sleepiness Scale, self‐reported), risk for SDB (based on (1) frequent snoring [self‐reported], (2) daytime sleepiness [Epworth Sleepiness Scale], (3) presence of hypertension or obesity [BMI])	Global cognitive function (Mini Mental State Examination [MMSE])	English or Spanish	Long sleep was inversely associated with performance on the MMSE. Short sleep was not associated with MMSE performance.
Ramos et al., 2016	8,676 H/L adults (ages 45–74 years, mean age of 56.5 years) from the Hispanic Community Health Study/Study of Latinos, 100% H/L	Sleep duration (weighted average of the difference between habitual wake and bedtimes, self‐reported)	Neurocognitive function (standardized scores for Word [Phonemic] Fluency [WF], brief‐Spanish English Verbal Learning Test [B‐SEVLT], and Digit Symbol Substitution [DSS] tests)	Participants’ preferred language	There were curvilinear inverted U‐shape associations between sleep duration and neurocognitive function. Participants with longer sleep duration had worse scores.
Insomnia					
Gallagher et al., 2022	846 older adults for the cross‐sectional analysis (mean age of 77.96 years ± 5.35 years); 308 participants for the longitudinal analysis, 9.81% H/L (9.45% H/L cognitively normal sample, 13.30% H/L CIND sample, 7.52% H/L dementia sample)	Insomnia symptoms and excessive sleep/sluggishness (sleep‐related questions from the Neuropsychiatric Inventory Questionnaire [NPI‐Q], informant‐reported)	Stage of cognitive decline (determined by a multidisciplinary team using neurocognitive test scores and DSM‐III‐R and DSM‐IV criteria)	English or Spanish	Hispanic older adults with cognitive impairment not dementia (CIND) have increased risk for insomnia symptoms. Additionally, there may be an association between insomnia symptoms and increased risk for cognitive impairment.
Ramos et al., 2021	16 consecutive treatment naïve male patients with moderate to severe OSA (ages 40–76 years, mean age of 59.7 years ± 9.4 years), 73% H/L	Insomnia severity index (ISI), Epworth Sleepiness Scale (ESS), Pittsburgh Sleep Quality Index (PSQI), obstructive sleep apnea ([OSA], in‐laboratory video polysomnography or type‐three home sleep apnea test)	Cognitive function (computerized neurocognitive testing, go‐no‐go response inhibition test, Stroop Interference Test, catch game test, staged information processing speed test, verbal memory test, and non‐verbal memory test) hippocampal and gray matter volumes (brain MRI T1‐weighted images)	Participants'' preferred language (English or Spanish)	The insomnia severity index score and average oxygen levels during sleep had strong correlations with cognitive function.
Sleep disordered breathing					
Kaur et al., 2021	5,500 H/Ls adults and older adults from the Study of Latinos‐Investigation of Neurocognitive Aging (SOL‐INCA, ages 45–74 years, mean age of 62.98 years ± 8.05 years), an ancillary study of the Hispanic Community Health Study/Study of Latinos (HCHS/SOL), 100% H/L	Baseline sleep disordered breathing (Respiratory Event Index [REI], Apnea Risk Evaluation [ARES]), sleepiness (Epworth sleepiness scale, self‐reported), sleep duration (self‐reported), insomnia (Women's Health Initiative Insomnia Rating Scale [WHIIRS])	7‐year neurocognitive decline (neurocognitive testing at baseline, visit 1, and visit 2: (1) Six‐ITEM Screener (SIS; mental status); (2) Brief Spanish English verbal learning test (B‐SEVLT; verbal episodic learning and memory); (3) controlled oral word association (or word fluency [WF]; verbal fluency) test of the multilingual aphasia examination; and (4) Digit symbol subtest (DSS; processing speed)	Participants’ preferred language (English or Spanish)	The association between combined SDB/long sleep and declines in memory and global cognition was most pronounced in obese older adults. Among women, MetS status modified the association between long sleep/SDB and decline in global cognition.
Ramos et al., 2020	5,247 H/Ls (mean age of 63 years ± 8 years) from the Hispanic Community Health Study/Study of Latinos, 100% H/L	Baseline sleep‐disordered breathing ([SDB], Respiratory Event Index [REI], Apnea Risk Evaluation [ARES]), daytime sleepiness (Epworth sleepiness scale [ESS]), insomnia (Women's Health Initiative Insomnia Rating Scale [WHIIRS]), and sleep duration (Sleep Heart Health Study Sleep Habits Questionnaire)	7‐year neurocognitive decline (change in episodic learning and memory [B‐SEVLT‐Sum and SEVLT‐Recall], language [word fluency {WF}], processing speed [digit symbol substitution], and a cognitive impairment screener [Six‐Item Screener {SIS}])	Participants’ preferred language (English or Spanish)	Long sleep but not short sleep was associated with decline in episodic learning and memory but not processing speed, after adjusting for covariates. SDB, sleepiness, and insomnia were not associated with neurocognitive decline. Long sleep duration predicted 7‐year cognitive decline.
Obstructive sleep apnea					
Ramos et al., 2015	8,059 H/Ls (ages 45–74 years, mean age of 56.4 years) from the Hispanic Community Health Study/Study of Latinos, 100% H/L	Obstructive sleep apnea ([OSA], Apnea–Hypopnea Index [AHI], apnea risk evaluation [ARES]), subjective sleep data (Sleep Heart Health Study Sleep Habits Questionnaire and Epworth Sleepiness Scale)	Neurocognitive function (Word Fluency [WF] test, brief‐Spanish English Verbal Learning Test [SEVLT], and Digit Symbol Substitution [DSS] test)	Participants' preferred language (English or Spanish)	There was an association between the AHI and all 4 neurocognitive test scores. Female sex was a moderating factor between the AHI and WF, SEVLT‐sum, SEVLT‐recall, and DSS. There is a relationship between worse neurocognitive function and OSA across diverse H/L groups. Women were more likely than men to have lower neurocognitive function associated with OSA.
Sleep onset latency					
Agudelo et al., 2021	1,035 H/Ls (ages 45–64 years, mean age 55.2 ± 2.5 years) from the Hispanic Community Health Study/Study of Latinos), 100% H/Ls	Obstructive sleep apnea ([OSA], Respiratory Event Index [REI], Apnea Risk Evaluation [ARES]), sleep duration (actigraphy), sleep continuity (actigraphy), sleep‐onset latency (actigraphy), sleep maintenance efficiency (actigraphy), naps and sleep duration per nap (actigraphy), daytime sleepiness (Epworth Sleepiness Scale, [ESS]), sleep quality, use of sleep medications, insomnia (Insomnia Severity Index, [ISI])	Cognitive function (Six‐Item Screener [SIS], brief‐Spanish English Verbal Learning Test [B‐SEVLT], phonemic verbal fluency test [word fluency, WF], Digit Symbol Subtest [DSS], trail making test Part B [only at visit 2])	Participants' preferred language (English or Spanish)	Longer sleep‐onset latency was associated with declines in global cognitive function, verbal learning, and verbal memory. Longer sleep‐onset latency was also cross‐sectionally associated with verbal learning, verbal memory, and word fluency. Cognitive change was not found to be associated with sleep fragmentation, sleep duration, sleep continuity, daytime sleepiness, or insomnia symptoms.

Abbreviations: CIND, cognitive impairment, not demented; DSM, Diagnostic and Statistical Manual of Mental Disorders; H/L, Hispanic/Latin American individuals; MRI, magnetic resonance imaging; SDB, sleep disordered breathing.

### 3.3 Results of individual studies

Due to the variability in the sleep parameters and cognitive measures examined across the identified studies, results are presented and discussed based on the primary sleep problems or parameters investigated. When applicable, comparisons across studies were made based on how sleep and cognition were examined. In our review, a total of five sleep problems or parameters were identified as having been explored in relation to cognition in H/L individuals.

#### 3.3.1 Sleep duration

Five studies examined associations between sleep duration and cognition in H/L adults.^25^, ^26^, ^27^, ^28^, ^29^ One of five studies utilized sleep actigraphy to objectively measure sleep duration.^25^ The remaining four studies utilized self‐report measures of sleep duration^26^, ^27^, ^28^, ^29^; however, specific questions or questionnaires varied.

A study conducted by Ramos and colleagues^27^ explored the relationship between both short and long self‐reported sleep duration and global cognitive function. In a sample of 927 individuals (40 years of age or older) from the Northern Manhattan Study cohort, cognitive function and sleep duration were measured via the Mini Mental State Examination (MMSE;^30^ and self‐report, respectively. Findings revealed that longer sleep duration (≥9 h) was significantly and inversely associated with MMSE scores in a fully‐adjusted model (*β* = −0.06, *p *= 0.012); individuals who reported longer sleep duration demonstrated lower MMSE scores.^27^

Similarly, Ramos and colleagues^28^ examined the relationship between self‐reported sleep duration and cognitive function as measured by a test battery that included measures of verbal fluency, episodic memory, and speeded complex attention. The sample of 8676 individuals ranged from 45 to 74 years old and were recruited as part of HCHS/SOL. In contrast to the previous two studies, Ramos and colleagues (2016) asked participants for their usual sleep and wake times to determine sleep duration. A weighted average was then calculated for average sleep duration. Results from a fully adjusted quadratic model revealed an inverted U‐shaped relationship between sleep duration and cognitive function (*β* range: −0.0090 to −0.0030; *p*: 0.037 to 0.070). Specifically, participants with intermediate sleep duration (7–8.71 h) exhibited higher levels of cognitive function, while those with longer (greater than 8.71 h) and shorter (less than 7 h) sleep durations demonstrated poorer cognitive function.^28^

A separate study by Ramos and colleagues^29^ examined whether sleep duration predicted seven‐year neurocognitive decline in older H/L adults from the HCHS/SOL study. After controlling for sex, time interval between sleep assessments, global vascular risk score, symptoms of depression, and frequency of sleep medication use, longer sleep duration (> 9 h) was associated with declines in episodic learning and memory, phonemic fluency, and mental status (*β* range: −0.22 to −0.13, *p *< 0.050), while processing speed remained unaffected (*β* = 0.018, *p≥*.050).^29^

A study by Kaur and colleagues^26^ also investigated the relationship between cognitive decline and sleep phenotypes (e.g., sleep duration and combined sleep‐disordered‐breathing with sleep duration) while considering potential modifiers such as obesity. The study included a sample of 5500 participants, ages 45 to 74 years old, from the Study of Latinos‐Investigation of Neurocognitive Aging (SOL‐INCA), an ancillary study of the HCHS/SOL study. Older adults (ages 65–74 years) with obesity (body mass index [BMI] *≥*30kj/m^2^) who reported both long sleep duration and SDB showed greater decline in global cognition and memory compared to older adults who were not obese (*M* = −0.96, 95% CI: [−1.71,−0.22]).^26^

Furthermore, a study by Agudelo and colleagues^25^ investigated the associations between cognition and various sleep parameters, including sleep duration, using actigraphy‐derived sleep patterns rather than self‐report^25^ in a sample of 1,035 H/L adults, 45 to 64 years of age, from the HCHS/SOL cohort. In contrast to the aforementioned studies relying on self‐report measures to determine sleep duration, a significant association was only found between sleep duration and a measure of speeded attention (Trail Making Test Part A, *b* = ‐ 0.063, *p *< 0.05). However, no significant associations were found in any of the other cognitive tests administered (*b* range = ‐0.057 to 0.015, *p *> 0.05).

#### 3.3.2 Insomnia symptoms

Four studies investigated the association between insomnia symptoms and cognition.^25^, ^29^, ^31^, ^32^ Gallagher and colleagues^31^ examined informant‐reported symptoms of insomnia across stages of cognitive decline in older adults ages 70 years or older. Utilizing data from the Aging, Demographics, and Memory Study, a supplementary study of the Health and Retirement Study, the authors conducted both cross‐sectional (*n* = 846) and longitudinal analyses (*n* = 308). Participants were divided into groups based on cognitive diagnostic status: cognitively normal (CN); cognitive impairment, not demented (CIND); and dementia. These were determined by multidisciplinary consensus confidence based on standardized criteria and cognitive test scores. In the cross‐sectional analyses, adults with cognitive impairment, but not dementia, had a higher prevalence of insomnia symptoms as opposed to adults without cognitive impairment, as indicated by informant reports [*χ* ^2^ (16, *N* = 767) = 250.90, *p* < 0.001].^31^ Findings also revealed that differential patterns of insomnia symptoms across diagnostic status differed significantly by race/ethnicity [*χ* ^2^ (2, *N* = 767) = 7.051, *p* = 0.003]. Notably, insomnia symptoms were more prevalent for Black and Hispanic older adults in the CIND group in comparison to the CN group. Differences between the CIND group and the dementia group differed between Black and Hispanic older adults. For Black older adults, the dementia group had a lower prevalence of insomnia symptoms than the CIND group. In contrast, the prevalence of insomnia symptoms was similar between the two groups for Hispanic older adults. Additionally, in longitudinal analyses, insomnia symptoms were associated with increased risk for development of cognitive impairment [*χ* ^2^ (5, *N* = 192) = 35.37, *p* < 0.001]; older adults were more likely to convert to CIND from CN by Visit 2 if they reported insomnia symptoms at Visit 1.^31^ The authors concluded that Black and Hispanic older adults with CIND were at an increased risk for symptoms of insomnia. Also, individuals reporting insomnia symptoms may be at increased risk for later development of cognitive impairment.

In a separate study, Ramos and colleagues^32^ examined the relationship of Insomnia Severity Index (ISI) scores with cognitive functioning and brain volume in a sample of 16 males with OSA ranging from 40 to 76 years old. ISI scores were strongly correlated with attention [*r*(14) = −0.66, *p *= 0.015] and volume of the inferior parietal gyrus [*r*(14) = −0.75, *p *= 0.010], which is involved in attentional processes. Given these findings, the authors suggest potential frontal‐temporal network dysfunction based on the effects of insomnia.^32^

Similarly, Ramos and colleagues^29^ examined whether insomnia predicted seven‐year neurocognitive decline in older H/L adults from the HCHS/SOL study. Insomnia was measured via self‐report using a questionnaire developed from the Women's Health Initiative Insomnia Rating Scale.^33^ Results from this study did not identify a significant relationship between insomnia and cognitive performance (*β* range: −0.051 to 0.052, *p *> 0.05).^29^

Agudelo and colleagues^25^ also investigated the associations between cognition and various sleep parameters, including insomnia symptoms. However, when using the presence of insomnia symptoms as a reference, no significant association was found with cognitive performance (*β* range: −0.030 to 0.080, *p *> 0.05).^25^

#### 3.3.3 SDB

Four studies examined the relationship between SDB and cognition,^26^, ^29^, ^32^, ^34^ all of which utilized data from the HCHS/SOL. Kaur and colleagues^26^ investigated the relationship between cognitive decline and sleep phenotypes (e.g., SDB) while considering potential modifiers, like obesity. The study included a sample of 5,500 participants, ages 45 to 74 years old, from SOL‐INCA. As noted previously, results demonstrated that older adults (ages 65‐74 years) with obesity (BMI ≥ 30kj/m^2^) who reported both long sleep duration and SDB show greater decline in global cognition and memory compared to older adults who were not obese (marginal estimate = −0.96 [−1.71; −0.22]).^26^

Ramos and colleagues^34^ examined the relationship between OSA and cognitive function in a sample of 8,059 community‐dwelling H/L adults ages 45–74 years from the HCHS/SOL study, considering and adjusting for BMI as a covariate in the full linear regression model. Measurement of cognitive function included assessment of verbal learning and memory, phonemic fluency, attention, and visuoperceptual abilities. Results revealed a lack of association between poorer cognitive performance and OSA across diverse H/L heritage groups in fully adjusted models (*β* range: −0.005 to 0.003; *p *> 0.10). However, female sex was a moderating factor between the apnea–hypopnea index (AHI) and neurocognitive performance for age groups 45–74 and 45–54 (*β* range: −0.057 to ‐0.037, *p *< 0.01) ^34^; women were more likely than men to have lower neurocognitive performance associated with OSA.

Similarly, Ramos and colleagues^29^ examined whether SDB predicted 7‐year neurocognitive decline in older H/L adults from the HCHS/SOL study. However, there was no evidence of a significant relationship between SDB and cognitive decline (*β* range: −0.051 to 0.052, *p *> 0.05).^29^

In a separate study, Ramos and colleagues^32^ examined the relationship between AHI scores and average oxygen levels during sleep with cognitive performance and age‐related brain loss in a sample of 16 males with OSA ranging from 40–76 years old. The AHI was negatively associated with memory (*r* = −0.70, *p *= 0.01), and average oxygenation during sleep was positively associated with memory (*r* = 0.64), attention (*r* = 0.69), and global cognition (*r* = 0.57).^32^

#### 3.3.4 SOL

One study examined the relationship between SOL and cognitive decline in H/Ls. Agudelo and colleagues^25^ utilized a sample of 1,035 H/L adults, 45—64 years of age, from the HCHS/SOL cohort to investigate the relationship between actigraphy‐derived sleep patterns and cognitive decline. Findings from this study revealed that increased SOL was cross‐sectionally associated with poorer performance in measures of global cognitive function, verbal episodic learning and memory, and word fluency (*β* coefficients ranged from −0.0032 to −0.0021, *p *< 0.01). Increased SOL was also longitudinally associated with declines in verbal learning and memory and mental status after 7‐year follow‐up (*β* coefficients ranged from −0.0036 to −0.0023, *p *< 0.05). These findings suggest that actigraphy‐derived SOL is a predictor for cognitive change within a 7‐year follow up.

## 4 DISCUSSION

### 4.1 Summary of evidence

Given the heightened risk for cognitive decline and poor sleep health among H/Ls, the identification of modifiable risk factors is essential to inform the development of targeted interventions. Therefore, this systematic review aimed to examine the current literature on the associations between sleep disturbances and cognitive function in H/L adults. Overall, the results of our review reveal an equivocal association between sleep parameters and cognitive performance in H/Ls at this time, with the most compelling evidence supporting a relationship between sleep duration and cognitive abilities.

The outcomes of this review indicate that longer sleep duration, typically exceeding 9 h, is associated with diminished cognitive function. However, this effect was not uniform across cognitive domains. Prior research on this association corroborates these results, supporting an association between longer sleep duration and worse cognitive function.^12^, ^35^, ^36^, ^37^ One possible explanation for the relationship between long sleep duration and cognition is that individuals with long sleep duration are at increased risk for or may show symptoms of other health conditions such as diabetes mellitus, hypertension, cardiovascular disease, stroke, coronary heart disease, and/or obesity, which may influence sleep.^38^, ^39^, ^40^, ^41^, ^42^ Depression and low socioeconomic status may also play a role in this relationship as possible mediators.^43^ Indeed, these various factors appear to be highly prevalent in H/L communities.^44^, ^45^ Another related explanation is that individuals with longer sleep duration are more likely to live a sedentary lifestyle and less likely to engage in protective brain health behaviors. That is, when individuals spend more time sleeping, they are spending less time exercising, socializing, or engaging in cognitively demanding activities, which have been shown to be protective factors for cognitive function.^46^, ^47^ Other evidence suggests that short sleep is also associated with poorer cognitive function; however, this was not a consistent finding in the present review.^12^, ^35^, ^36^, ^37^ Interestingly, one study in the present review identified an inverted U‐shaped relationship between sleep duration and cognitive function; a finding supported to some extent by previous literature.^12^, ^35^ Inconsistencies in measurement and/or sampling may also contribute to these equivocal findings. Thus, additional research is necessary to elucidate the nature of these associations.

A single study examining SOL revealed an inverse relationship between SOL and overall cognitive function.^25^ Research in this area is limited; while there is some support from prior literature that shorter SOL is associated with better cognitive performance,^48^, ^49^ Schmutte and colleagues^48^ report that their results should be interpreted with caution due to the use of an experimental sleep measure that was made specifically for their study and had not been validated. Thus, more research in this area is necessary.

Regarding insomnia symptoms, there was evidence to support a link between insomnia symptoms and cognitive decline, where individuals with symptoms of insomnia were more likely to have cognitive impairment. Previous research has shown that insomnia symptoms are associated with poorer overall cognitive function and increased risk for cognitive impairment.^50^, ^51^

In the context of SDB, three of four studies demonstrated significant associations with cognitive decline.^26^, ^29^, ^32^, ^34^ While prior research offers some support for SDB as a risk factor for cognitive impairment,^52^ the relationship between changes in oxygen saturation levels and cognitive function is less clear.^53^ Notably, a previous study by Cohen‐Zion and colleagues^53^ suggested that cognitive decline was associated with increases in a respiratory disturbance index. This is consistent with our present findings. However, this relationship did not hold in the study's more inclusive model, which also included variables of SDB, oximetry, sleep, and subjective report. Findings of this same study also suggested that cognitive decline was associated primarily with daytime sleepiness. Therefore, there is a possibility that a shared pathway exists between these two factors, and future research is needed to investigate this further. Other potential contributors to this relationship, such as snoring or waking up in the middle of the night due to apneic events, should also be explored.

Also, in the realm of SDB, the negative impact of OSA on cognition was evident in both studies included in this review.^32^, ^34^ The results of these studies substantiate its negative impact on cognition, such that lower oxygen levels during sleep were linked to reduced cognitive functioning in H/L adults. Previous research also indicates OSA as a risk factor for Alzheimer's disease.^12^, ^54^ However, it is possible that OSA‐related consequences (e.g., intermittent hypoxia, sleep fragmentation, reduced slow‐wave sleep, and intrathoracic pressure swings) may be driving these findings.

A review of systematic reviews and meta‐analyses on sleep disorders and cognitive function^55^ found compelling evidence for relationships between various sleep disorders and cognitive performance. Similar to the results of the present review, Kong and colleagues^55^ reported associations between long sleep duration and declines in cognitive performance. Notably, they reported strong evidence for short sleep and poorer cognitive performance. However, in the present review only one out of five studies examining sleep duration identified an inverted U‐shaped association between sleep duration and cognition, with both short and long sleep durations being related to poorer cognitive performance. Regarding insomnia, Kong and colleagues reported associations between insomnia and various cognitive domains (e.g., working memory, episodic memory, and problem‐solving). In contrast, the present study yielded mixed findings, which hindered the ability to draw overarching conclusions regarding this relationship, especially regarding specific cognitive domains. In terms of SDB, Kong and colleagues reported associations between SDB, including OSA, with higher risk of cognitive impairment. While one study from the present review identified associations between combined long SD and SBD with greater declines in global cognition and memory, methodologies and findings were mixed amongst the remaining studies. Although SOL was not investigated in depth in the review by Kong and colleagues, they reported its impact on cognition and exacerbation of cognitive impairment. This is consistent with the results of the present study, which found that increased SOL was associated with declines in cognitive performance. Nevertheless, given that only one study was identified examining SOL's impact on cognition, further research is necessary to confirm this relationship.

It is also important to note that overlapping relationships may exist between different sleep parameters and cognition. For example, a potential confounding factor for long sleep duration's association with cognition is sleep apnea.^38^ Similarly, individuals with insomnia will experience shorter sleep duration. Furthermore, potential confounding factors outside of other sleep factors may influence the observed relationships in this review, including chronic stress, depression, anxiety, medication use, chronic pain, and timing of physical activity in relation to sleep onset.

The equivocal associations between sleep and cognitive performance in H/Ls observed in the present review may also be partly explained by unidentified mechanisms of resilience among H/Ls in the United States. Despite having higher incidences of cardiovascular risk factors, research on cardiovascular outcomes in H/Ls in the United States has shown lower incidences of negative cardiovascular outcomes and better life expectancy than expected compared to non‐Hispanic White individuals, referred to as the “Hispanic Paradox.”^56^ A similar paradoxical trend may be present in the relationships between sleep and cognition in H/Ls. Future research is needed to determine if such a trend exists and to identify possible mechanisms of resilience.

The results of this review provide valuable insights that may support future research on modifiable risk factors and sleep interventions for cognitive decline. Understanding and mitigating these risk factors can improve the quality of life and well‐being of older adults and reduce the societal burden of these conditions.

### 4.2 Limitations

Our review had several limitations. First, the present study describes results on studies involving sleep duration, insomnia symptoms, SDB, and SOL. However, other important sleep parameters, such as sleep efficiency, wake after sleep onset, sleep architecture, and napping, were not discussed due to the scarcity of such topics in H/Ls. This highlights a critical gap in the literature for this population. Additionally, the studies included in the review had methodological heterogeneity such as in how sleep parameters were operationalized (i.e., criteria used) and measured (i.e., objective vs. subjective), precluding direct comparisons. Most studies relied on self‐report for sleep parameters that may contribute to ambiguous findings. Variability in the definitions of sleep parameters and covariate adjustments also threaten the generalizability of findings. Additionally, the studies incorporated into this review predominantly employed cross‐sectional analyses, limiting our capacity to infer causal relationships between sleep and cognition. Cohort effects from the cross‐sectional studies may also introduce confounding factors into our review's findings. Moreover, it presents a substantial impediment to effectively discerning how this relationship evolves across the aging process, as it lacks multiple time points. Sleep patterns are dynamic and fluctuate throughout the lifespan due to external factors such as work or family obligations. Thus, effective assessment of sleep's influence on cognitive ability requires consideration of time‐dependent relationships and the presence of period(s) of vulnerability where optimal sleep quality and quantity may have greater effects. A significant portion of the studies included in the synthesis of this review used data from the HCHS/SOL cohort, increasing the risk of bias and limiting the generalizability of the results. This may result in over‐representation of data from this cohort and contribute disproportionately to the overall results and thus, not provide a well‐rounded view of the evidence. Furthermore, the variation in the representation of H/L individuals in some studies raises concerns about the applicability of these results to the broader H/L population. While seven of eight studies included in this review had at least 68% H/Ls in their sample, one study had less than 10% H/Ls in their sample.^31^ Lastly, the wide array of types of sleep disturbances also poses a challenge in terms of comprehensive coverage in the identification of studies for this review. It is possible that not all articles examining sleep disturbances in H/Ls were identified with the key terms that were utilized for the initial search.

### 4.3 Future directions

Further research is needed to explore the causal relationships, underlying mechanisms, and shared pathways among sleep, cognition, and other health conditions (e.g., cardiovascular disease, depression, and anxiety). Additionally, future research in H/Ls is needed for other sleep parameters such as sleep efficiency, wake after sleep onset, and sleep architecture, as well as understanding the effects of other sleep‐related factors, such as frequency and duration of naps. The challenge presented in the observed heterogeneity in sleep and cognitive measures may be resolved if future research utilized a standardized method of assessing these parameters. This would improve the effective comparison of results between studies. Furthermore, future endeavors could benefit from the use of objective measures of sleep in their studies to enhance our understanding of the sleep‐cognition relationship. Lastly, our review underscores a pressing demand for longitudinal research examining the relationship between sleep and cognition in H/Ls. Such longitudinal studies are essential not only for a deeper exploration of this relationship but also for the creation of more diverse datasets to support future research endeavors. The inclusion of a wider range of datasets can foster increased diversity in perspectives and research methodologies, ultimately contributing to a richer and more nuanced comprehension of the intricate relationship between sleep parameters and cognitive abilities.

### 4.4 Conclusion

This review suggests a link between sleep variables and cognition in H/L adults, with the most robust evidence supporting a link between sleep duration and cognitive abilities. These results suggest that interventions targeting sleep hygiene and quality could potentially reduce the risk of cognitive decline and improve cognition in this population. However, further longitudinal studies are necessary to validate these findings and explore potential contributing factors such as depressive symptoms, anxiety symptoms, cardiovascular disease, and hypoxia. Additionally, environmental and sociocultural factors should also be explored such as the presence of light and noise pollution in predominantly H/L neighborhoods. Moreover, the current state of the literature in this population only allows us to make preliminary conclusions and hinders the generalizability of the findings in the present review; the field of sleep and cognition in H/Ls remains inadequately explored, underscoring the need for further investigation.

## AUTHOR CONTRIBUTIONS

**Candice A. Alfano**: Conceptualization; writing—review and editing; supervision. **Kasey J. Escamilla**: Conceptualization; methodology; validation; formal analysis; investigation; data curation; writing—original draft; writing—review and editing; visualization. **Alexa S. Gonzalez**: Validation; investigation; data curation; writing—original draft; writing—review and editing. **Michelle N. Martinez**: Validation; investigation; writing—original draft; writing—review and editing. **Luis D. Medina**: Conceptualization; methodology; writing—review and editing; supervision.

## CONFLICT OF INTEREST STATEMENT

The authors declare no conflicts of interest. Author disclosures are available in the supporting information.

## DATA SHARING

Data extraction tables are available from the corresponding author upon reasonable request.

## USE OF GENERATIVE AI AND AI‐ASSISTED TECHNOLOGIES

Generative AI was not used in the preparation and creation of this paper.

## Supporting information



Supporting Information
